# Structural Basis for the Recognition in an Idiotype-Anti-Idiotype Antibody Complex Related to Celiac Disease

**DOI:** 10.1371/journal.pone.0102839

**Published:** 2014-07-30

**Authors:** Anna Vangone, Safwat Abdel-Azeim, Ivana Caputo, Daniele Sblattero, Roberto Di Niro, Luigi Cavallo, Romina Oliva

**Affiliations:** 1 Department of Chemistry and Biology, University of Salerno, Fisciano, Salerno, Italy; 2 Kaust Catalysis Center, King Abdullah University of Science and Technology, Thuwal, Saudi Arabia; 3 European Laboratory for the Investigation of Food-Induced Diseases (ELFID), University Federico II, Naples, Italy; 4 Department of Health Sciences and Interdisciplinary Research Center of Autoimmune Diseases (IRCAD), University of Eastern Piedmont, Novara, Italy; 5 Department of Immunology, University of Pittsburgh, Pittsburgh, Pennsylvania, United States of America; 6 Department of Sciences and Technologies, University “Parthenope” of Naples, Naples, Italy; Jacobs University Bremen, Germany

## Abstract

Anti-idiotype antibodies have potential therapeutic applications in many fields, including autoimmune diseases. Herein we report the isolation and characterization of AIM2, an anti-idiotype antibody elicited in a mouse model upon expression of the celiac disease-specific autoantibody MB2.8 (directed against the main disease autoantigen type 2 transglutaminase, TG2). To characterize the interaction between the two antibodies, a 3D model of the MB2.8-AIM2 complex has been obtained by molecular docking. Analysis and selection of the different obtained docking solutions was based on the conservation within them of the inter-residue contacts. The selected model is very well representative of the different solutions found and its stability is confirmed by molecular dynamics simulations. Furthermore, the binding mode it adopts is very similar to that observed in most of the experimental structures available for idiotype-anti-idiotype antibody complexes. In the obtained model, AIM2 is directed against the MB2.8 CDR region, especially on its variable light chain. This makes the concurrent formation of the MB2.8-AIM2 complex and of the MB2.8-TG2 complex incompatible, thus explaining the experimentally observed inhibitory effect on the MB2.8 binding to TG2.

## Introduction

**Figure 1:**
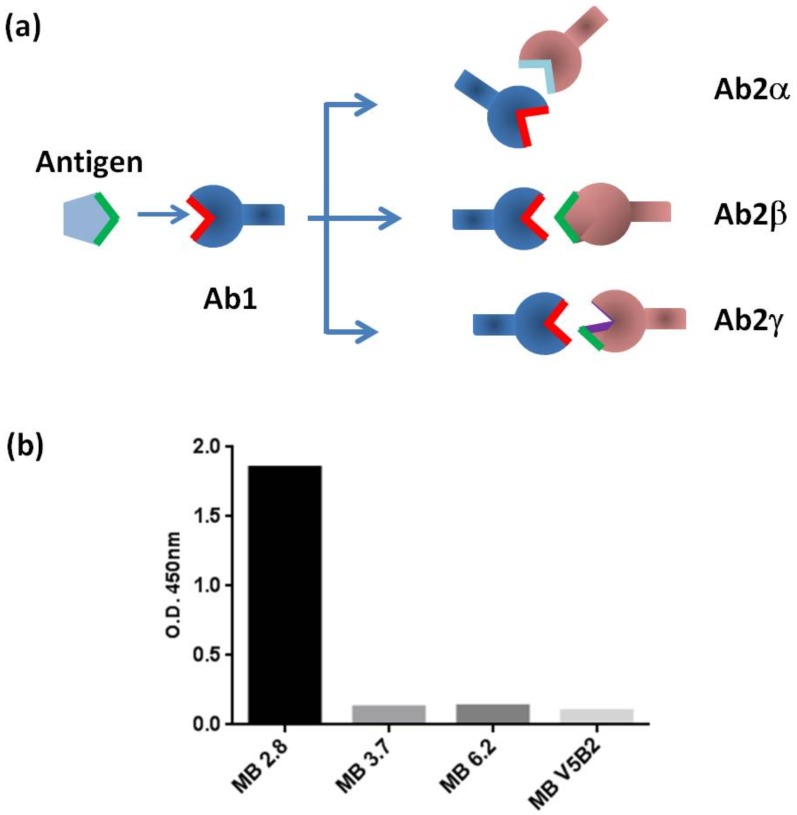
Scheme of the idiotypic network and specificity of the AIM2 response. **a**) The idiotypic network. An antigen, Ag, is recognized by its antibody Ab1. The Ab1 becomes itself an antigen eliciting the production of anti-antibodies Ab2. This response can be divided into: i) an antigen-non inhibitable group (Ab2α), ii) an antigen-inhibitable group bringing an “internal image” of the antigen (Ab2β), and iii) an antigen-inhibitable group due to steric hindrance with the antigen binding-site (Ab2γ). **b**) Specificity of AIM2 response. Immunoreactivity of phage expressed AIM2 scFv was tested by ELISA against MB2.8 (used for selection) and 3 others scFv-Fc. MB3.7 is a celiac-derived anti-TG2 antibody while MB6.2 and MBV5B2 are control antibodies using the VH5 gene for VH Chain.

It has been long established that the structural basis for antigen (Ag) recognition by antibodies (Abs) relies on the length and sequence variability of the six Ab complementary determining regions (CDRs) [1]. Based on the combinatorial origin of this limited region, made by about 70 residues, antibodies are able to recognize almost an infinite variety of antigens, from small organic molecules to proteins. Interestingly, antibodies can be antigenic themselves, being recognized by other antibodies and thus creating a network, through which immunoglobulins expression may be controlled. According to the “idiotypic network hypothesis” [2], under specific immunological conditions, antigen stimulation leads to the production of idiotype antibodies (termed Ab1) against Ag, characterized by specific antigenic-determinants (the “idiotopes”). The unique structure of the Ab1 antigen-binding site can generate in turn the production of a series of anti-idiotype antibodies, termed Ab2s, which are directed against the Ab1 antigenic-determinants (Figure 1a) and may or may not represent an image of the original Ag. Finally, anti-anti-idiotypes antibodies (Ab3s) can be induced by the presence of Ab2, which may have binding capabilities similar to those of Ab1, thus recognizing the original antigen. An anti-idiotype antibody can be classified as: i) *“Ab2-alpha”*(Ab2α); ii) *“Ab2-beta”* (Ab2β); iii)*“Ab2-gamma”* (Ab2γ), on the basis of their ability to inhibit the binding of Ab1 to the original antigen (see Figure 1a) [3], [4].

Several experimental evidences have demonstrated the crucial role played by the idiotypic Ab1-Ab2-Ab3 network in the regulation of immune response to both external and self antigens [4], [5]. In recent years, extensive research has been devoted to the possible therapeutic application of anti-idiotype antibodies. Ab2s have been the basis for developing new generation vaccines [6], [7] and novel therapeutic approaches for the treatment of tumours [7], [8], such as breast cancer [9], [10], colorectal carcinoma [11], melanoma and ovarian lymphoma [12], [13]. They have also been suggested for the design of anti-HIV strategies for AIDS [14], [15] and as potent anticoagulants to restore normal haemostasis [16].

The idiotypic network has also been shown to have a fundamental role in the autoimmune diseases. While the factors leading to the onset of the autoimmune response remain obscure, the idiotypic disregulation is now indeed recognized as a major mechanism for autoimmunity [17]–[22]. Deficient idiotypic regulation of autoantibodies has been considered a contributing factor for a number of autoimmune diseases [22], such as systemic lupus erythematosus (SLE) [19], autoimmune thyroiditis [17], systemic vasculitis [18] and the Guillain-Barrè syndrome [21]. Furthermore, it has been demonstrated that autoimmune patients show a large ratio of autoantibody to anti-idiotype concentration whereas this ratio is small in healthy controls [20]. *In vivo* studies have indicated that anti-idiotypic antibodies might be able to downregulate the autoantibodies, making the use of Ab2s very promising in the study and treatment of autoimmune diseases. In type 1 diabetes, for example, it has been recently shown that anti-idiotypes may play a protective role in the immune response, by preventing the autoantibody from binding its antigen [23]. Another intriguing application of Ab2 is in creating animal models to study autoimmunity by inducing it in animals through the usage of pathogenic idiotypes of autoantibodies. Following immunization with Ab1 and production of Ab2s, the animals may also develop Ab3s, having original autoantibodies properties and being associated with the respective serological and clinical manifestations of the disease [4], [24].

One of the most common diseases with autoimmune features that suffers from a lack of animal models is celiac disease (CD). CD is characterized by the presence of specific antibodies recognizing an endomysial autoantigen identified as type 2 transglutaminase (TG2) [25]. The antibody level against TG2 increases upon exposure to gluten, and decreases during the course of a gluten-free diet [26]. We have recently [27] found clear evidence that a specific anti-idiotypic response could be induced in mouse by ectopically expressing an anti-TG2 antibody derived from CD patients [28] and cross-reactive with the mouse protein. Furthermore, by ELISA assays, this response was able to compete with immobilized TG2 antigen for anti-TG2 binding, suggesting a binding restricted or close to the original antibody CDR region [27].

Here we report the isolation and characterization of an anti-idiotypic antibody that we named AIM2 (Anti-Idiotype Mouse 2) from a mouse (mouse 2, in Di Niro et al. [27]) giving the highest binding specificity towards the celiac autoantibody MB2.8 expressed in the same mouse. To characterize the interaction between the anti-TG2 antibody (Ab1-MB2.8) and its anti-idiotype (Ab2-AIM2), here we present the 3D model we obtained for the MB2.8-AIM2 complex by docking simulations. We applied a new protocol to the analysis and selection of representative decoys, based on the conservation of inter-residue contacts at the interface [29]–[33]. We have previously proposed intermolecular contact maps (i.e. maps where a black dot is present at the crossover of two interacting residues) as fingerprints of the interface in protein complexes [29]–[31]. They report the crucial information in a ready-to-read form and allow to easily and intuitively discriminate between similar and different binding solutions [30]. Furthermore, we have shown that intermolecular contact maps, together with the measure of the conservation of inter-molecular contacts, can be used to analyse docking model ensembles [29] and to reliably extract from them the native-like solutions [31], [32].

Interestingly, as a result of this analysis, a clearly-preferred solution emerged, that we thoroughly characterized in its static and dynamic features and compared with the six available experimental structures of Ab1-Ab2 complexes [14], [16], [34]–[37].

## Materials and Methods

### Anti-idiotype single chain (sc) Fv isolation

The phage display Ab library used for selection was derived from spleen lymphocytes isolated from mouse N.2 [27], previously characterized to have an anti-idiotypic response to the *in vivo*-expressed recombinant scFv-Fc MB2.8 specific for TG2. The library was constructed according to standard procedure [38]. Panning was performed in immunotubes coated with human scFv-Fc antibody MB2.8 by overnight incubation at 4°C as previously reported [38]. The panning procedure was repeated twice. 96 random clones were selected and the phages from single colonies were grown in 96-well plates. A positive clone (AIM2) was identified by phage ELISA and the V genes was sequenced and the VH and VL families, as well as the gene segments used, were assessed by screening using the IMGT/V-QUEST tool in theIMGT, the ImMunoGeneTics information system http://imgt.cines.fr. The positive scFv AIM2 was converted into a scFv-Fc format first by subcloning into the pMB-SV5 [39] vector containing the human IgG1 Hinge-CH2-CH3 domain. The recombinant antibody was finally subcloned into pUCOE vector [40] for expression and purification.

### Molecular modeling

#### Abs modeling

The variable domain structures of MB2.8 and Ab2-mouse AIM2 were modeled by the RosettaAntibody Server [41], [42], using the default full refinement protocol option. The PDB codes of the templates for Ab1 MB2.8, used in the simulations, are as follows (sequence identity is indicated in parentheses): 3NCJ for the heavy chain framework (95%) and 1T3F for the light chain (98%); 1DFB for L1 (100%), 1DFB for L2 (86%), 2NY4 for L3 (60%); 2H32 for H1 (90%), 2XWT for H2 (94) and no template for H3 (re-modeled *de novo* using a kinematic loop modeling algorithm developed in a Rosetta protocol). The PDB codes of the templates for Ab2 AIM2 are as follows (sequence identity is indicated in parentheses): 1MH5 for the heavy-chain framework (97%) and 1AY1 for the light chain (97%); 1AY1 for L1 (100%), 1SEQ for L2 (100%), 1AY1 for L3 (78%); 1IQW for H1 (90%), 1IQW for H2 (100%) and no template for H3 (re-modeled *de novo*, see above). Length and canonical structures of the loops, reported in Table 1, were assigned by DIGIT [43].

**Table 1:** Length and canonical structure (CS) of the CDRs in the modelled antibodies and in the corresponding templates.

**AIM2**					
**loop**	**length**	**predicted-CS**	**template**	**length**	**CS**
L1	10	kappa 1	1AY1	10	kappa 1
L2	7	kappa 1	1SEQ	7	kappa 1
L3	9	kappa 1	1AY1	9	kappa 1
H1	5	1	1IQW	5	1
H2	17	2	1IQW	17	2
H3	9	N/A	No template	N/A	N/A
**MB2.8**					
**loop**	**length**	**predicted-CS**	**template**	**length**	**CS**
L1	11	kappa 2	1DFB	11	kappa 2
L2	7	kappa 1	1DFB	7	kappa 1
L3	11	kappa O	2NY4	11	kappa 8
H1	5	1	2H32	5	1
H2	17	2	2XWT	17	2
H3	12	N/A	No template	N/A	N/A

#### Molecular docking

The obtained models were then used for Ab1/Ab2 protein-protein docking simulations, performed by the ClusPro 2.0 server [44]. In all the simulations, the Ab2 residues that did not fall into the CDRs were masked, using the ClusPro Antibody Mode option [45]. Differently, for MB2.8 two situations were explored. In the first one, indicated in the following as ‘CDR-directed’, all residues but the CDR ones were masked, to have only the CDRs available for interaction; in the second one, indicated as ‘blind’ docking, all the MB2.8 residues were considered on an equal basis, masking only Ab2 CDR residues.

#### Decoys analysis

The interface in the docking decoys and the X-ray structure 1DVF [34] (one of the available experimental Ab1-Ab2 structures) were analyzed, visualized and compared by the COCOMAPS [30] and CONS-COCOMAPS [29] web tools. The representative structures of the obtained clusters for each docking simulation (22 for the CDR-directed and 30 for the ‘blind’ one) were ranked with CONSRANK [31], [32]. Finally, a local structural similarity between the 3D model of Ab2-AIM2 and the X-ray structure of the original antigen [46], [47] of Ab1-MB2.8 was performed by RASMOT 3D PRO [48], ProBis 2012 [49] and LGA [50] web tools with default parameters.

#### Molecular dynamics simulations

The molecular dynamics (MD) simulations were performed using the GROMACS software (version 4.5.3) [51] with the Amber99SB-ILDN force field [52]. All systems were slightly relaxed using 50 steps of steepest descent minimizer. The systems were then immersed in an explicit water box of TIP3P model [53], which extended at least 10 Å away in each direction from any atom of the complex. 20160 and 23094 water molecules were added to the models and to 1DVF, respectively. Ten and three chloride ions were added to the models and to 1DVF to neutralize the positive charges, as needed for the particle mesh Ewald calculation [54] of the long-range electrostatic interactions, while a cut-off of 10 Å was used for van der Waals and short-range electrostatic interactions. 500 steps of steepest descent minimization were performed to remove bad contacts with the solvent. All bonds involving hydrogen atoms were constrained by the LINCS algorithm [55]. Equilibration of the solvent and ions around the complexes with position constraints of the heavy atoms was performed for 2 ns in the NVT ensemble, followed by 2 ns in the NPT ensemble. NVT simulations were carried out using the velocity rescaling thermostat (V-rescale) [56] and the NPT ones using Parrinello-Rahman barostat [57]. MD production simulations were performed in the NPT ensemble for 100-ns for M1-CDR (see below for the models nomenclature) and 1DVF, and for 20-ns for M2-CDR and M1-blind. RMSD, gyration radius and energy values were calculated with standard GROMACS tools. To assess the convergence of the 1DVF and M1-CDR trajectories, we split them into two halves, and we calculated the root mean square inner product (RMSIP) between the first 10 eigenvectors of PCA analysis on the two halves [58], [59]. Furthermore, we evaluated the cosine content of the first 10 eigenvectors using the Hess' approach [60], [61].

## Results and Discussion

### AIM2 isolation and characterization

In a previous work [27], we have found clear evidence that an anti-idiotypic response is induced in mice expressing *in vivo* antibodies isolated from CD patients directed to TG2 (antibody MB2.8). In the present work, a phage scFvs library was constructed from lymphocytes isolated from mouse N.2 that showed the strongest anti-idiotypic response to the MB2.8 molecule. The scFvs library was then selected against recombinant MB2.8 antibodies and, after screening, a positive anti-idiotype clone (AIM2) was identified by phage ELISA. The specificity of the recognition was confirmed by testing it both as scFv displayed on phage and as scFv-Fc recombinant antibody against other antibodies. AIM2 was shown to recognize specifically only MB2.8 (Figure 1b) while it did not bind other TG2-specific antibodies isolated from CD patients [28] or control antibodies belonging to the same gene family (VH5). Sequencing of the V genes showed that the VL chain used the IGKV4-55*01 V region with a CDR3 of 9 amino acids (89–97 definition), while the VH region used the IGHV1S13*01 with a 8 amino acids long CDR3 sequence (95–102 definition). AIM2 DNA and protein sequences are reported in the Supplementary Materials (Sequences S1 and S2 in File S1).

### Molecular modeling

#### Modeling of Ab1-MB2.8 and Ab2-AIM2

To obtain the structure of the complex between the celiac autoantibody MB2.8 and its anti-idiotype AIM2, by docking simulations, we first modelled the variable Fab region of the two antibodies with the RosettaAntibody server [41], [42]. Sequence identity with the templates used to model the framework regions is above 95% (see Materials and Methods). Modelling of H3 loops, which can be challenging, is performed by RosettaAntibody *de novo*, using the well-known Rosetta fragment-based approach [62]. Extensive benchmarking showed that it can reliably predict the H3 loop conformation in most of the cases, but for very long loops (above 17 aa) [63]. Antibody non-H3 CDRs are instead well known to present a limited number of main-chain conformations, also named “Canonical Structures (CSs)”, based on their length and the presence of key-residues at specific positions [64]. This makes the reliable modeling of such regions an easy task, provided that the right templates are selected. In Table 1, for each CDR, we report, when applicable, the length, the predicted CS, the PDB ID of the template used to model it and the length and CS of the corresponding loop in the template. From Table 1, it is clear that selected templates share with the CDRs to model length and CS. The only exception is CDR-L3 in MB2.8. As its sequence fails by canonical classification, it was modelled based on the structure of 2NY4 CDR-L3, presenting a “kappa 8” CS [65] and sharing with it the length (11 residues) and the presence of a key proline at position 95.

It is worth noting here that RosettaAntibody was specifically developed to obtain highly-refined antibody models, to be also used in docking simulations, and extensively benchmarking indeed confirmed the utility of obtained models in docking simulations [63].

#### Docking simulations: CDR-directed and ‘blind’

Protein-protein docking simulations were performed by ClusPro 2.0 [44]. In our ClusPro Ab1-Ab2 simulations, Ab1-MB2.8 acts as the antigen (i.e. the recognized molecule), while Ab2-AIM2 acts as the antibody (i.e the recognizing molecule). Therefore, according to the ClusPro criterions [44], Ab2-AIM2 was fixed and all its residues not falling into the CDR were masked.

**Figure 2:**
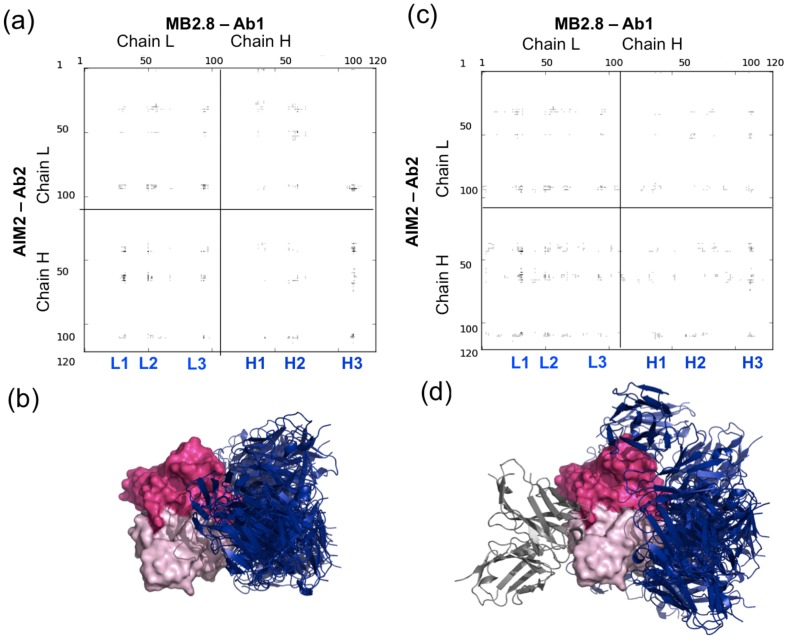
Comparison between the top ‘CDR-directed’ and ‘blind’ docking solutions. (**a**) and (**c**): the CONS-COCOMAPS consensus maps calculated for the 22 models from the CDR-directed docking simulations (**a**) and for the 30 models from the ‘blind’ ones (**c**). Labels have been added for the MB2.8 CDR loops L1-L3 and H1-H3. (**b**) and (**d**): Pymol [70] visualization of the representative models of the top ten clusters in both CDR-directed (**c**) and ‘blind’ docking simulations (**d**), after superimposition of Ab1-MB2.8. The MB2.8's light chain is colored in pink, its heavy chain is colored in hotpink. All the models using the MB2.8 CDR region for the binding have AIM2 colored in blue, while AIM2 is colored in silver in the 7^th^ solution of the blind simulation.

As the experimental data showed that anti-idiotype antibodies elicited in mouse strongly compete with the original TG2 antigen for the MB2.8 binding (suggesting that the MB2.8 binding sites for TG2 and for AIM2 at least partially overlap) [27], in a first set of simulations (CDR-directed) the non-CDR regions of MB2.8 were also masked. The 2000 obtained CDR-directed decoys resulted in 22 clusters, with the top-ranked ones highly populated (152 and 118 decoys for the first two clusters, over the total of 2000 decoys considered). The representative structures for each of the 22 obtained clusters (i.e. the one at the center of the cluster) were analyzed with the CONS-COCOMAPS [29] server. The consensus map (Figure 2a) showed at a glance the similarity among these 22 docking solutions, since the dark spots converge in defined regions, located at the crossover of specific CDR loops, mainly Ab1-MB2.8 L1, L2, L3 and H3 and Ab2-AIM2 L3, H1, H2 and H3. In Table S1 in File S1, the detailed list of the inter-residue contacts conserved in at least 25% of the models is reported, as determined by CONS-COCOMAPS, with the corresponding conservation rates.

Consistent results were obtained from a second set of ‘blind’ simulations, where Ab1-MB2.8 was not forced to interact through its CDR residues (i.e., no Ab1-MB2.8 residue was masked during the docking simulation). Although these ‘blind’ simulations gave clusters with a lower population, as compared with the CDR-driven ones (86 vs. 152 decoys for the most populated cluster), they converged towards a similar binding mode. The representative models of the ten top-ranked clusters, among the 30 obtained, still pointed to the CDR region of MB2.8, with the only exception of one model (ranked 7^th^ according to the cluster population, see Figure 2d), where Ab2-AIM2 pointed to an Ab1-MB2.8 region opposite to the CDR. A solution that is not biologically plausible when the complete antibodies are considered. This was actually the only obtained model incompatible with the binding of the full antibody. The similarity between the results from the CDR-driven and blind simulations was apparent both from the 3D representation of the 10 top ranked models and from the comparison of the consensus maps reported in Figure 2. In fact, although the ensemble of top ranked ‘blind’ solutions presented some additional spots, the darkest ones still corresponded to those found by the CDR-driven simulations.

#### Selection of representative models

ClusPro ranks the obtained docking solutions based on the population of the corresponding decoys cluster [44], and the population of a decoys cluster is indeed commonly used as a criterion to assess the reliability of a docking solution. For the selection of the most “promising” solutions, in addition, we used CONSRANK, a consensus method we recently developed for ranking docking models, based on the conservation of inter-residue contacts in the decoys ensemble [29], [31], [32]. In particular, CONSRANK calculates the conservation rate for each inter-residue contact in a models ensemble and then ranks models according to their ability to match the most frequently observed contacts, which can be regarded as key contacts for the interaction. A very good performance was found when applying CONSRANK to over 100 docking targets from three different benchmarks [31], [32]. Herein, CONSRANK was applied to both the 22 CDR-driven and 30 blind models separately, and on the ensemble of the 52 CDR-driven and blind models together. Overall, the four top CONSRANK solutions, when all the 52 ClusPro centroids are considered, correspond to the ClusPro CDR-driven models 1 and 2 (hereafter M1-CDR and M2-CDR) and to the ‘blind’ models 1 and 3 (M1-blind and M3-blind, see Table 2), thus supporting the ClusPro population-based ranking. The only discrepancy between the ClusPro and the CONSRANK analysis concerns M2-blind, which is ranked low by CONSRANK. The consistency between the ClusPro clustering analysis and the CONSRANK consensus analysis indicates that the centroids of the most populated clusters have an interface that is characterized by the contacts most frequently observed in the whole set of centroids. In other words, the most populated cluster is also the one able to better match a large number of highly-conserved contacts among all the analysed solutions.

**Table 2:** Top 10 ClusPro models for the CDR-directed and “blind” simulations, with corresponding ClusPro population and ranking position by CONSRANK.

	**ClusPro Population**	**CONSRANK-CDR rank**	**CONSRANK-all rank**
M1-CDR	152	2	3
M2-CDR	118	1	2
M3-CDR	67	10	8
M4-CDR	65	11	9
M5-CDR	58	9	10
M6-CDR	38	8	5
M7-CDR	38	16	18
M8-CDR	32	13	26
M9-CDR	32	21	31
M10-CDR	32	3	11
	**ClusPro Population**	**CONSRANK-blind rank**	**CONSRANK-all rank**
M1-blind	86	1	4
M2-blind	82	7	16
M3-blind	72	2	1
M4-blind	69	13	38
M5-blind	44	5	20
M6-blind	43	4	23
M7-blind	41	14	43
M8-blind	37	19	41
M9-blind	34	29	49
M10-blind	33	9	34

Supported by the convergent ClusPro and CONSRANK results, we focused our attention on the four top solutions according to the CONSRANK-all ranking mode. However, since M2-CDR and M3-blind are extremely similar (the RMSD of the whole complex being only 0.79 Å), only M2-CDR was retained for further analyses, together with M1-CDR and M1-blind. These three models present a similar binding mode, reflected by the low values of the RMSD, within ≈10 Å, for the backbone superposition of Ab1-MB2.8, upon best superposition of Ab2-AIM2 (see Table 3). It is worth reminding here that standard in the docking field is considering such RMSD values good enough for having that two structures are a reasonable approximation one of the other [66], [67]. However, the three models clearly differ in the details of the interaction, such as the number of inter-molecular H-bonds and of residues at the interface (Table 4). In particular, M1-CDR presents a larger interface (839.6 Å^2^) compared to the other two models, in line with Ab1-Ab2 of known structure (Table S2 in File S1). Both based on its higher interface area and of its lower pair-wise Lrmsd_bb values, M1-CDR seems thus to be the best choice as a representative model for the MB2.8-AIM2 complex.

**Table 3:** RMSD values calculated on the backbone of Ab1-MB2.8 upon best Ab2-AIM2 superposition.

	M1-CDR	M2-CDR	M1-blind
M1-CDR	-	9.2	6.5
M2-CDR		-	10.4
M1-blind			-

**Table 4:** Comparison of some interface features of the three selected MB2.8-AIM2 models and of the 1DVF structure, by the COCOMAPS server [30].

	Interface area (Å^2^)	# Interface ress (Ab2/Ab1)	# H-bonds	# Salt bridges	# phil-phil	# phob-phob	%Polar
M1-CDR	840	45(23/22)	16	4	53(64)	1	48.2
M2-CDR	638	37(21/16)	9	2	44(52)	7	51.2
M1-blind	772	45(22/23)	13	4	53(65)	0	48.2
1DVF	816	46(21/25)	11	1	43(59)	3	55.5

#### Detailed analysis of M1-CDR and Comparison with an Ab1-Ab2 X-ray structure

A detailed analysis of M1-CDR interface was performed by the COCOMAPS web tool (Table 4). The complex is stabilized by all six CDRs of both MB2.8 and AIM2, with the V_L_ of MB2.8 and the V_H_ of AIM2 being predominant in such contacts. In particular, MB2.8 and AIM2 are aligned along an ideal axis perpendicular to their CDRs, with the MB2.8 light chain facing the AIM2 heavy chain and *viceversa*. The interface area is 840 Å^2^, with a percentage of polar residues at the interface of approximately 48%.

**Figure 3:**
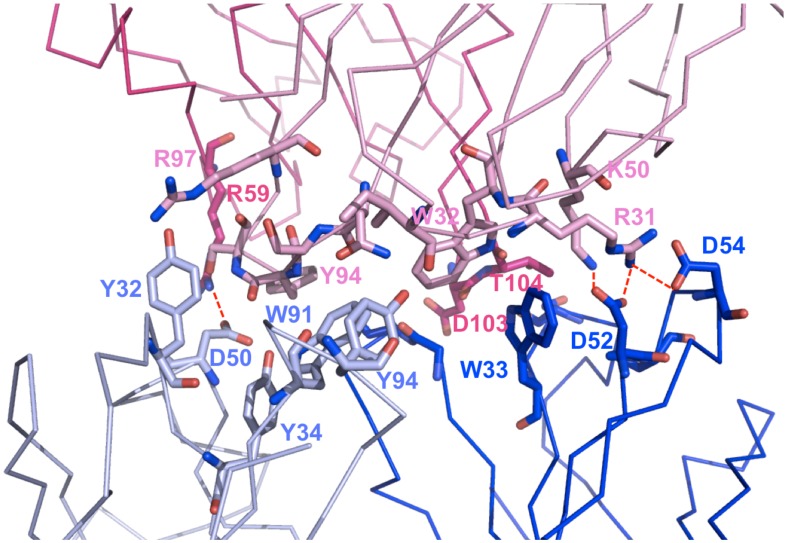
M1-CDR interface. A surface representation of the MB2.8-AIM2 interface in M1-CDR. The MB2.8 light and heavy chains are colored in pink and hotpink, respectively, while the AIM2 light and heavy chains are colored in lightblue and blue. All residues at the interface that are more than half buried upon complex formation and all residues involved in inter-molecular salt bridges are shown as sticks and labelled.

Both the AIM2 and MB2.8 interfaces involved in the interaction present a high percentage of hydrophilic residues. Using a cut-off distance of 5 Å to define two atoms in contact, 53 over the total 64 inter-residue contacts occur between two hydrophilic residues. The complex is also stabilized by a network of 16 hydrogen bonds, including 4 salt-bridges involving residues Arg31L (loop L1) and Arg59H (loop H2) of MB2.8 and Asp54H (loop H2) and Arg31L (loop L1) of MB2.8 (L1) and Asp50L (loop L2), Asp52H and Asp54H (loop H2) of AIM2. Light and heavy chains of both antibodies are equally represented in the H-bonds network, with a slight predominance of the AIM2 heavy chain (participating in 9 H-bonds and 3 salt-bridges). Eleven residues in both AIM2 and MB2.8 have their accessible surface area (ASA) reduced by more than 50% upon complex formation (see Table 5 and Figure 3). These include most of the residues involved in H-bonds and also several aromatic residues, such as AIM2 Trp33H and MB2.8 Trp32L, involved in a π stacking interaction, AIM2 Trp91L and MB2.8 Tyr94L, mainly involved in hydrophobic interactions, and AIM2 Tyr32L, Tyr34L and Tyr94L, giving H-bonds with MB2.8 Ile29L, Asn92L and Arg97L. In Figure 3, all the residues at the interface, which are more than half buried upon complex formation, and all those involved in inter-molecular salt bridges are shown.

**Table 5:** M1-CDR residues that are more than 50% buried upon complex formation.

AIM2-Ab2		MB2.8-Ab1	
Residue	Buried ASA (%)	Residue	Buried ASA (%)
Asp52a_H	98.68	Lys50_L	95.15
Pro52b_H	96.59	Arg31_L	84.59
Asp50_L	86.02	Asp103_H	83.25
Tyr34_L	79.87	Ser93_L	81.08
Trp33_H	76.94	Thr104_H	78.37
Ser31_H	69.35	Tyr94_L	75.50
Trp91_L	69.26	Trp32_L	68.97
Tyr94_L	68.38	Asn92_L	67.17
Ser97_H	67.50	Ile29_L	59.97
Gly96_H	63.66	Ser95_L	58.76
Tyr32_L	58.22	Arg97_L	50.13

**Figure 4:**
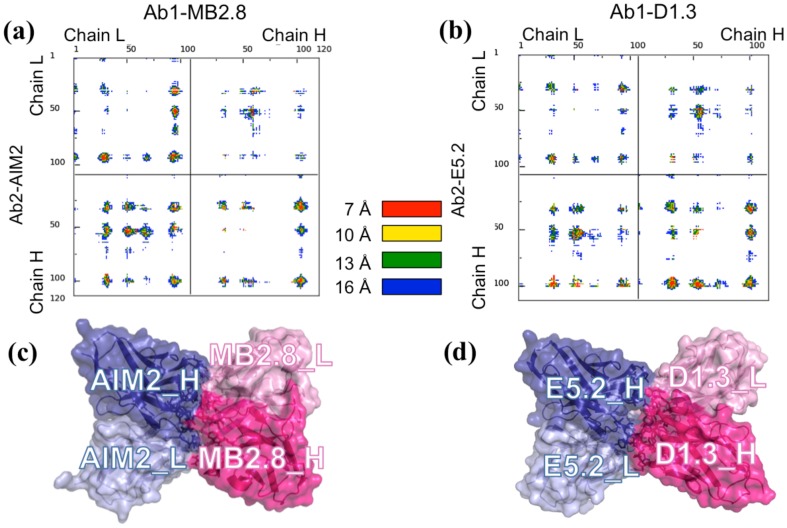
Comparison between M1-CDR and the X-ray structure of the D1.3-E5.2 Ab1-Ab2 complex (1DVF). (**a**) and (**b**): the distance range contact maps by COCOMAPS [30], calculated for the MB2.8-AIM2 model (**a**) and the experimental structure of E5.2/D1.3 complex (PDB code: 1DVF) (**b**). The dots at the crossover of two residues are colored in red, yellow, green and blue if any pair of atom is closer than 7, 10, 13 and 16 Å, respectively. (**c**) and (**d**): Pymol [70] visualization of MB2.8-AIM2 model (**c**) and the experimental structure E5.2-D1.3 (**d**). The color code is the same in both figures: the Ab1 light and heavy chains are colored in light and dark blues, respectively; the Ab1 light and heavy chains are colored in light and dark pink, respectively. Labels have been added for the Ab1's and Ab2's light and heavy chains.

Remarkably, the above Ab1-Ab2 binding solution shows a striking similarity with the binding mode adopted by four of the six known experimental Ab1-Ab2 structures [14], [16], [34]–[37], specifically with the crystal structures corresponding to the PDB IDs: 1DVF [34], 1PG7 [16], JMB94 (our code, this structure was published in J Mol Biol in 1994 [35] and never deposited in the PDB) and, to a smaller extent, 1IAI [36]. 80% of the M1-CDR heavy atoms can be superimposed to 1DVF, 1PG7 and JMB94 with a RMSD below 5 Å, and to 1IAI with a RMSD of ≈7 Å. However, the experimental structure most similar to the M1-CDR model, in terms of RMSD, is 1DVF, a complex between an anti-hen-egg-white lysozyme antibody, Ab1-D1.3, and its anti-idiotypic antibody Ab2-E5.2 [34]. 79% of M1-CDR heavy atoms (2756 out of 3481) can indeed be superimposed to corresponding atoms in 1DVF with an RMSD value of only 3.2 Å. A detailed comparison with 1DVF is thus reported in the following. The sequence identity of the D1.3-E5.2 complex with the MB2.8-AIM2 complex is about 65% for both the L and H chains of the Ab1s and 66% and 48% for the alignment of the L and H chains, respectively, of the two Ab2s. In Figure 4, the contact maps of both the MB2.8-AIM2 M1-CDR and the 1DVF crystallographic structure [34] are reported. From their comparison, it is apparent that the MB2.8-AIM2 model has similar binding interactions to those in 1DVF. In fact, both complexes are preferentially stabilized by contacts between: Ab1 L1, L3 and H2 loops and the light chain of Ab2, and Ab1 L1, L2, L3 and H3 loops and the heavy chain of Ab2. Similar contacts are also observed for the X-ray 1PG7, JMB94 and 1IAI structures, as can be seen from corresponding contact maps in Figure S1 in File S1, although in 1IAI the interaction between the Ab1 chain L and Ab2 chain H is less significant. The interface area of M1-CDR and 1DVF is also very similar (840 Å^2^ for the MB2.8-AIM2 and 816 Å^2^ for the D1.3-E5.2, see Table 4). Analogously to the MB2.8-AIM2 complex, in D1.3-E5.2 about half (46%) of the buried surface upon complex formation is polar, most of the inter-molecular contacts occur between hydrophilic residues (43 out of 59) and the complex is stabilized by many inter-molecular H-bonds (11, including one salt bridge). Overall, 23 residues (9 belonging to E5.2 and 14 to D1.3) have more than 50% surface buried upon complex formation. They include polar/charged residues involved in inter-molecular H-bonds, but also several aromatic residues (see Table S3 in File S1), giving different types of interaction.

#### Molecular Dynamics Simulations of M1-CDR

**Figure 5:**
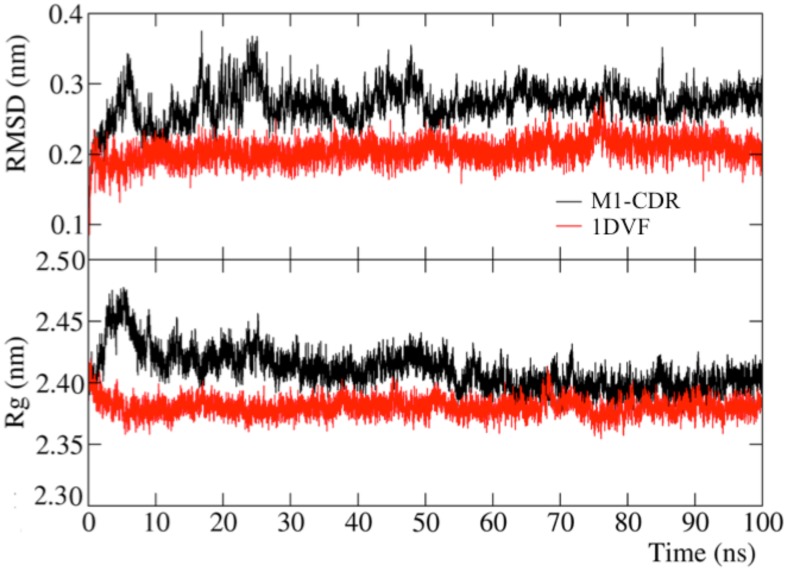
RMSD fluctuation and gyration radius. **Top**: RMSD fluctuation of the Cα atoms of M1-CDR and 1DVF from the starting geometry along the 100-ns long MD trajectory. **Bottom**: Gyration radius, Rγ, of M1-CDR and 1DVF along the 100-ns long MD trajectory.

To test the overall stability of M1-CDR, we performed a 100-ns long molecular dynamics simulation. For comparison, we performed a similar simulation for 1DVF Ab1-Ab2 complex [30]. The good convergence of the simulations is reflected by the high root mean square inner product (RMSIP) values, 0.77 for M1_CDR and 0.81 for 1DVF, calculated between the first 10 principal component vectors of the two halves of the trajectories [58], [59], and by the low cosine content of the first 10 principal component vectors (Table S4 in File S1) [60], [61]. Both systems are similarly stable in terms of the RMSD of the Cα atoms from the starting structure (values around 2-3 Å), with marginally higher oscillations for M1-CDR, see Figure 5 (top). A rather stable behavior is also found when the focus is on the radius of gyration Rγ, a property linked to the molecular volume and compactness, see Figure 5 (bottom). The only difference is in an initial marginal fluctuation of the Rγ of M1-CDR from 2.40 to 2.46 nm, which however relaxes to the initial value along the whole trajectory. This analysis thus indicates that M1-CDR does not undergo severe conformational rearrangements, and, under dynamic conditions, it behaves similarly to the 1DVF complex. On the other hand, the MD simulations outline the relative instability of the other top ranked models, M1-blind and especially M2-CDR. Indeed, for these models the RMSD on the Cα shows values above 8 and 5 Å, respectively, within the first 20 ns of simulation, while their gyration radius largely fluctuates, raising to 2.55–2.60 nm (see Figure S2 in File S1).

#### Searching for structural similarities between AIM2 (Ab2) and TG2 (Ag)

The last step in the characterization of an Ab1-Ab2 complex is to identify possible structural similarities between the anti-idiotype antibody and the original antigen (TG2). As an experimental structure for the TG2-MB2.8 complex (the corresponding Ag-Ab1 complex) is currently unavailable, we only searched for a possible local structural similarity between Ab2 and the original Ab1 antigen TG2. Two web tools used to the aim, RASMOT 3D PRO [48] and ProBis 2012 [49], were unable to detect any significant similarity.

However, another web server, LGA, found a significant structural similarity between the whole TG2 N-terminal domain (open form [47]) and the AIM2 heavy chain, superimposing the Cα atoms of 94 corresponding residues with a RMSD of 2.77 Å and a quality score (LGA_S) of 3.27 (2.0 being the threshold for weak alignments). This is not surprising, as the N-terminal domain of TG2 is characterized by an Immunoglobulin-like beta-sandwich fold. In Figure S3 in File S1, the TG2-to-AIM2 structural correspondence found by LGA is shown, together with the MB2.8-AIM2 binding mode in M1-CDR. From it, it appears that, in principle, MB2.8 could bind to the TG2 N-terminal domain, analogously to how it binds to AIM2, without significant steric hindrance. However, the composition of the AIM2 (Ab2) CDRs mainly involved in the MB2.8 binding is quite different from that of the structurally corresponding TG2 segments. Therefore, even if this structural correspondence was the basis for a similar binding mode to the MB2.8 antibody, the anti-idiotype AIM2 antibody could hardly be described as a molecular mimic of the TG2 antigen.

## Conclusions

The high level expression in a mouse model of a cross-reactive anti-TG2 antibody (MB2.8) leads to a specific and prolonged anti-idiotypic response [27]. Here we report the isolation and computational characterization of the interaction between the celiac anti-TG2 antibody MB2.8 and the anti-idiotype antibody, AIM2, that was elicited in the mouse model. The specific monoclonal Ab2 AIM2 was isolated after construction of a scFv antibody phage library from a mouse showing an anti-idiotypic immune response to the CD derived MB2.8 antibody and validated for specificity to the original target.

Molecular modeling simulations were performed on the MB2.8-AIM2 complex and, as a result, a quite clear picture emerged about its structural features. The different solutions clearly pointed to a preferred interface area. The model we selected, M1-CDR, is very well representative of the different solutions found, at least in terms of interface contacts. This observation increases our confidence in the obtained model. Furthermore, an *a posteriori* comparison between the selected model and the available experimental structures of Ab1-Ab2 complexes showed that it is strikingly similar to most of them, and particularly similar to the complex between Ab1-D1.3 and Ab2-E5.2 (PDB ID: 1DVF), both in its static and dynamics features, despite the obvious differences in sequence. This unexpected result represents, in our opinion, an independent validation of the obtained model.

Our detailed interface analysis shows that Ab2-AIM2 is directed against the Ab1-MB2.8 CDR region, especially on V_L_. This makes the contemporary formation of the MB2.8-AIM2 (Ab1-Ab2) complex and of the MB2.8-TG2 complex (Ab1-TG2) incompatible, thus explaining the experimental data showing a strong inhibitory effect of the anti-idiotype antibodies elicited in mouse on the MB-2.8 binding to TG2 [27]. The search we performed for possible structural similarities between Ab2-AIM2 and original MB-2.8 antigen, i.e. TG2, detected a structural similarity between the N-terminal domain of TG2 in its open form and the AIM2 heavy chain. However, the highly different amino acid composition of the structurally correspondent segments does not allow to depicting the anti-idiotype AIM2 antibody as a molecular mimic of the TG2 antigen. Therefore, as AIM2 is an anti-idiotype antibody which binds the Ab1 (MB2.8) on its antigen-binding site, without apparently carrying any internal image of the original antigen (TG2), we suggest its classification as an Ab2γ, that is also the category in which most of the Ab2s having an experimental structural characterization to date fall [14], [16], [35], [37]).

Due to the crucial involvement of the idiotypic network in the autoimmune diseases and the promising therapeutic applications, the detailed model we present for Ab2-AIM2 and for its interaction with Ab1-MB2.8 could be the basis for new possible therapeutic strategies, such as the design of humanized antibodies for the inhibition of TG2 binding by autoantibodies in celiac disease. Indeed, several experimental evidences have highlighted the possible pathogenic role of anti-TG2 autoantibodies, as a consequence of their interaction with TG2 [68], [69]. Anti-idiotype antibodies have already been shown *in vivo* to be able to down-regulate autoantibodies, for instance in type-1 diabetes, by preventing them from binding to the autoantigen [23].

To our knowledge, this is the first report of docking simulations performed to predict the structure of an Ab1-Ab2 complex. Due to their complexity and despite their great biomedical interest, this class of protein complexes has been to date under-characterized from a structural point of view, both experimentally and computationally. We proposed here a protocol for the analysis of docking results, mainly based on the conservation of the contacts at the interface, which can be applied to the study of both Ab1-Ab2 and other protein complexes. Furthermore, we hypothesize that the similarity we found between our model and Ab1-Ab2 experimental structures may be more general and likely also apply to other Ab1-Ab2 complexes. It could be speculated that, analogously to the ability of antibodies at recognizing an almost infinite number of molecules, based on a surprisingly invariant structural basis [64], also the recognition between antibodies may be achieved based on a limited set of binding solutions. If confirmed, this will make the desirable task of modeling antibody-to-antibody complexes a rather affordable one.

## Supporting Information

File S1 **Supplementary Materials.** Sequence S1, AIM2 DNA sequence. Sequence S2, AIM2 protein sequence. Table S1, list of inter-residue contacts in the 22 CDR solutions. Table S2, interface features of the six structure-known Ab1-Ab2 complexes. Table S3, list of 1DVF residues more than 50% buried upon complex formation. Table S4, cosine content of the first ten MD eigenvectors calculated for 1DVF and M1_CDR. Figure S1, intermolecular contact maps generated by COCOMAPS for the M1-CDR model and the six experimental structures available for Ab1-Ab2 complexes. Figure S2, RMSD fluctuation and Gyration radius, Rγ, for M1-CDR, M2-CDR, M1-blind and 1DVF along the MD simulations. Figure S3, LGA superimposition between the N-terminal domain of TG2 in its open form and the heavy chain of AIM2.
(DOCX)

## References

[1] Wu TT, Kabat EA (1970) An analysis of the sequences of the variable regions of Bence Jones proteins and myeloma light chains and their implications for antibody complementarity. J Exp Med 132: 211–250. 5508247 10.1084/jem.132.2.211 PMC2138737

[2] Jerne NK (1974) Towards a network theory of the immune system. Ann Inst Pasteur Imm 125C: 373–389. 4142565

[3] Dalgleish AG, Kennedy RC (1988) Anti-idiotypic antibodies as immunogens - idiotype-based vaccines. Vaccine 6: 215–220. 3048008 10.1016/0264-410x(88)90213-7

[4] Abu-Shakra M SY (2007) Idiotypes and antiidiotypes. Autoantibodies. pp. 69–76.

[5] Pan Y, Yuhasz SC, Amzel LM (1995) Antiidiotypic antibodies - biological function and structural studies. Faseb J 9: 43–49. 7821758 10.1096/fasebj.9.1.7821758

[6] Wilks D, Dalgleish AG (1992) Anti-idiotypic therapeutic strategies in HIV infection. Molecular and cell biology of human diseases series 1: 283–308. 1341647 10.1007/978-94-011-2384-6_10

[7] Bhattacharya-Chatterjee M, Chatterjee SK, Foon Ka (2000) Anti-idiotype vaccine against cancer. Immunol Lett 74: 51–58. 10996628 10.1016/s0165-2478(00)00249-2

[8] Warncke M, Buchner M, Thaller G, Dodero A, Bulashevska A, et al (2011) Control of the specificity of T cell-mediated anti-idiotype immunity by natural regulatory T cells. Cancer Immunol Immun 60: 49–60. 10.1007/s00262-010-0918-x PMC3029831 20848095

[9] Ladjemi MZ, Chardes T, Corgnac S, Garambois V, Morisseau S, et al (2011) Vaccination with human anti-trastuzumab anti-idiotype scFv reverses HER2 immunological tolerance and induces tumor immunity in MMTV.f.huHER2 (Fo5) mice. Breast Cancer Res 13: R17. 21294885 10.1186/bcr2826 PMC3109586

[10] Mohanty K, Saha A, Pal S, Mallick P, Chatterjee SK, et al (2007) Anti-tumor immunity induced by an anti-idiotype antibody mimicking human Her-2/neu. Breast Cancer Res Tr 104: 1–11. 10.1007/s10549-006-9391-9 17004107

[11] Chong G, Bhatnagar A, Cunningham D, Cosgriff TM, Harper PG, et al (2006) Phase III trial of 5-fluorouracil and leucovorin plus either 3H1 anti-idiotype monoclonal antibody or placebo in patients with advanced colorectal cancer. Ann Oncol 17: 437–442. 16311275 10.1093/annonc/mdj090

[12] Park HJ, Neelapu SS (2008) Developing idiotype vaccines for lymphoma: from preclinical studies to phase III clinical trials. British J Haematol 142: 179–191. 10.1111/j.1365-2141.2008.07143.x PMC2744838 18422783

[13] Weiner LM, Dhodapkar MV, Ferrone S (2009) Monoclonal antibodies for cancer immunotherapy. Lancet 373: 1033–1040. 19304016 10.1016/S0140-6736(09)60251-8 PMC2677705

[14] Bryson S, Julien JP, Isenman DE, Kunert R, Katinger H, et al (2008) Crystal structure of the complex between the F(ab)' fragment of the cross-neutralizing anti-HIV-1 antibody 2F5 and the F(ab) fragment of its anti-idiotypic antibody 3H6. J Mol Biol 382: 910–919. 18692506 10.1016/j.jmb.2008.07.057

[15] Burioni R, Mancini N, De Marco D, Clementi N, Perotti M, et al (2008) Anti-HIV-1 response elicited in rabbits by anti-idiotype monoclonal antibodies mimicking the CD4-binding site. PloS One 3: e3423. 18923648 10.1371/journal.pone.0003423 PMC2565497

[16] Eigenbrot C, Meng YG, Krishnamurthy R, Lipari MT, Presta L, et al (2003) Structural insight into how an anti-idiotypic antibody against D3H44 (anti-tissue factor antibody) restores normal coagulation. J Mol Biol 331: 433–446. 12888350 10.1016/s0022-2836(03)00735-6

[17] Dietrich G, Varela FJ, Hurez V, Bouanani M, Kazatchkine MD (1993) Selection of the expressed B cell repertoire by infusion of normal immunoglobulin G in a patient with autoimmune thyroiditis. Eur J Immunol 23: 2945–2950. 8223872 10.1002/eji.1830231133

[18] Jayne DR, Esnault VL, Lockwood CM (1993) Anti-idiotype antibodies to anti-myeloperoxidase autoantibodies in patients with systemic vasculitis. J Autoimmun 6: 221–226. 8388692 10.1006/jaut.1993.1019

[19] Jost CR, Kurucz I, Jacobus CM, Titus JA, George AJ, et al (1994) Mammalian expression and secretion of functional single-chain Fv molecules. J Biol Chem 269: 26267–26273. 7929344

[20] Lundkvist I, van Doorn PA, Vermeulen M, Brand A (1993) Spontaneous recovery from the Guillain-Barre syndrome is associated with anti-idiotypic antibodies recognizing a cross-reactive idiotype on anti-neuroblastoma cell line antibodies. Clin Immunol Immunop 67: 192–198. 10.1006/clin.1993.1064 8500266

[21] Lundkvist I, van Doorn PA, Vermeulen M, van Lint M, van Rood JJ, et al (1989) Regulation of autoantibodies in inflammatory demyelinating polyneuropathy: spontaneous and therapeutic. Immunol Rev 110: 105–117. 2676844 10.1111/j.1600-065x.1989.tb00029.x

[22] Zanetti M (1986) Idiotypic regulation of autoantibody production. Crit Rev Immunol 6: 151–183. 3089685

[23] Tzioufas AG, Routsias JG (2010) Idiotype, anti-idiotype network of autoantibodies: pathogenetic considerations and clinical application. Autoimmun Rev 9: 631–633. 20478412 10.1016/j.autrev.2010.05.013

[24] Shoenfeld Y (1996) Common infections, idiotypic dysregulation, autoantibody spread and induction of autoimmune diseases. J Autoimmun 9: 235–239. 8738968 10.1006/jaut.1996.0029

[25] Dieterich W, Ehnis T, Bauer M, Donner P, Volta U, et al (1997) Identification of tissue transglutaminase as the autoantigen of celiac disease. Nat Med 3: 797–801. 9212111 10.1038/nm0797-797

[26] Maki M, Sulkanen S, Collin P (1998) Antibodies in relation to gluten intake. Dig Dis 16: 330–332. 10207216 10.1159/000016885

[27] Di Niro R, Sblattero D, Florian F, Stebel M, Zentilin L, et al (2008) Anti-idiotypic response in mice expressing human autoantibodies. Mol Immunol 45: 1782–1791. 17996305 10.1016/j.molimm.2007.09.025

[28] Marzari R, Sblattero D, Florian F, Tongiorgi E, Not T, et al (2001) Molecular dissection of the tissue transglutaminase antoantibody response in celiac disease. J Immunol 166: 4170–4176. 11238668 10.4049/jimmunol.166.6.4170

[29] Vangone A, Oliva R, Cavallo L (2012) CONS-COCOMAPS: a novel tool to measure and visualize the conservation of inter-residue contacts in multiple docking solutions. BMC Bioinformatics 13 Suppl 4S19. 10.1186/1471-2105-13-S4-S19 PMC3434444 22536965

[30] Vangone A, Spinelli R, Scarano V, Cavallo L, Oliva R (2011) COCOMAPS: a web application to analyse and visualize contacts at the interface of biomolecular complexes. Bioinformatics 27: 2915–2916. 21873642 10.1093/bioinformatics/btr484

[31] Oliva R, Vangone A, Cavallo L (2013) Ranking multiple docking solutions based on the conservation of inter-residue contacts. Proteins 81: 1571–1584. 23609916 10.1002/prot.24314

[32] Vangone A, Cavallo L, Oliva R (2013) Using a consensus approach based on the conservation of inter-residue contacts to rank CAPRI models. Proteins 81: 2210–2220. 24115176 10.1002/prot.24423

[33] Abdel-Azeim S, Chermak E, Vangone A, Oliva R, Cavallo L (2014) MDcons: Intermolecular contact maps as a tool to analyze the interface of protein complexes from molecular dynamics trajectories. BMC Bioinformatics 15 Suppl 5S1. 10.1186/1471-2105-15-S5-S1 PMC4095001 25077693

[34] Braden BC, Fields BA, Ysern X, DallAcqua W, Goldbaum FA, et al (1996) Crystal structure of an Fv-Fv idiotope - Anti-idiotope complex at 1.9 angstrom resolution. J Mol Biol 264: 137–151. 8950273 10.1006/jmbi.1996.0629

[35] Evans SV, Rose DR, To R, Young NM, Bundle DR (1994) Exploring the mimicry of polysaccharide antigens by anti-idiotypic antibodies. The crystallization, molecular replacement, and refinement to 2.8 A resolution of an idiotope-anti-idiotope Fab complex and of the unliganded anti-idiotope Fab. J Mol Biol 241: 691–705. 8071993 10.1006/jmbi.1994.1544

[36] Ban N, Escobar C, Garcia R, Hasel K, Day J, et al (1994) Crystal-structure of an idiotype antiidiotype fab complex. Proc Natl Acad Sci USA 91: 1604–1608. 8127852 10.1073/pnas.91.5.1604 PMC43211

[37] Bentley GA, Boulot G, Riottot MM, Poljak RJ (1990) Three-dimensional structure of an idiotope anti-idiotope complex. Nature 348: 254–257. 1700305 10.1038/348254a0

[38] Sblattero D, Bradbury A (2000) Exploiting recombination in single bacteria to make large phage antibody libraries. Nat Biotechnol 18: 75–80. 10625396 10.1038/71958

[39] Di Niro R, Ziller F, Florian F, Crovella S, Stebel M, et al (2007) Construction of miniantibodies for the in vivo study of human autoimmune diseases in animal models. BMC Biotechnol 7: 46. 17678525 10.1186/1472-6750-7-46 PMC1963447

[40] Boscolo S, Mion F, Licciulli M, Macor P, De Maso L, et al (2012) Simple scale-up of recombinant antibody production using an UCOE containing vector. Nat Biotechnol 29: 477–484. 10.1016/j.nbt.2011.12.005 22226921

[41] Sircar A, Kim ET, Gray JJ (2009) RosettaAntibody: antibody variable region homology modeling server. Nucleic Acids Res 37: W474–479. 19458157 10.1093/nar/gkp387 PMC2703951

[42] Lyskov S, Chou FC, Conchuir SO, Der BS, Drew K, et al (2013) Serverification of molecular modeling applications: the Rosetta Online Server that Includes Everyone (ROSIE). PloS One 8: e63906. 23717507 10.1371/journal.pone.0063906 PMC3661552

[43] Chailyan A, Tramontano A, Marcatili P (2012) A database of immunoglobulins with integrated tools: DIGIT. Nucleic Acids Res 40: D1230–1234. 22080506 10.1093/nar/gkr806 PMC3245095

[44] Comeau SR, Gatchell DW, Vajda S, Camacho CJ (2004) ClusPro: a fully automated algorithm for protein-protein docking. Nucleic Acids Res 32: W96–99. 15215358 10.1093/nar/gkh354 PMC441492

[45] Brenke R, Hall DR, Chuang GY, Comeau SR, Bohnuud T, et al (2012) Application of asymmetric statistical potentials to antibody-protein docking. Bioinformatics 28: 2608–2614. 23053206 10.1093/bioinformatics/bts493 PMC3467743

[46] Liu SP, Cerione RA, Clardy J (2002) Structural basis for the guanine nucleotide-binding activity of tissue transglutaminase and its regulation of transamidation activity. Proc Natl Acad Sci USA 99: 2743–2747. 11867708 10.1073/pnas.042454899 PMC122418

[47] Pinkas DM, Strop P, Brunger AT, Khosla C (2007) Transglutaminase 2 undergoes a large conformational change upon activation. PLoS Biol 5: 2788–2796. 10.1371/journal.pbio.0050327 PMC2140088 18092889

[48] Debret G, Martel A, Cuniasse P (2009) RASMOT-3D PRO: a 3D motif search webserver. Nucleic Acids Res 37: W459–464. 19417073 10.1093/nar/gkp304 PMC2703991

[49] Konc J, Janezic D (2012) ProBiS-2012: web server and web services for detection of structurally similar binding sites in proteins. Nucleic Acids Res 40: W214–221. 22600737 10.1093/nar/gks435 PMC3394329

[50] Zemla A (2003) LGA: A method for finding 3D similarities in protein structures. Nucleic Acids Res 31: 3370–3374. 12824330 10.1093/nar/gkg571 PMC168977

[51] Hess B, Kutzner C, van der Spoel D, Lindahl E (2008) GROMACS 4: Algorithms for highly efficient, load-balanced, and scalable molecular simulation. J Chem Theory Comp 4: 435–447. 10.1021/ct700301q 26620784

[52] Lindorff-Larsen K, Piana S, Palmo K, Maragakis P, Klepeis JL, et al (2010) Improved side-chain torsion potentials for the Amber ff99SB protein force field. Proteins 78: 1950–1958. 20408171 10.1002/prot.22711 PMC2970904

[53] Jorgensen WL, Duffy EM, Tiradorives J (1983) Comparison of Simple Potential Functions for Simulating Liquid Water. J Chem Phys 79: 926–935.

[54] Darden T, York D, Pedersen L (1993) Particle Mesh Ewald - an N.Log(N) Method for Ewald Sums in Large Systems. J Chem Phys 98: 10089–10092.

[55] Hess B, Bekker H, Berendsen HJC, Fraaije JGEM (1997) LINCS: A linear constraint solver for molecular simulations. J Comp Chem 18: 1463–1472.

[56] Bussi G, Donadio D, Parrinello M (2007) Canonical sampling through velocity rescaling. J Chem Phys 126: 014101. 17212484 10.1063/1.2408420

[57] Parrinello M, Rahman A (1981) Polymorphic Transitions in Single-Crystals - a New Molecular-Dynamics Method. J Appl Phys 52: 7182–7190.

[58] Amadei A, Ceruso MA, Di Nola A (1999) On the convergence of the conformational coordinates basis set obtained by the essential dynamics analysis of proteins' molecular dynamics simulations. Proteins 36: 419–424. 10450083

[59] de Groot BL, van Aalten DM, Amadei A, Berendsen HJ (1996) The consistency of large concerted motions in proteins in molecular dynamics simulations. Biophys J 71: 1707–1713. 8889148 10.1016/S0006-3495(96)79372-4 PMC1233640

[60] Hess B (2000) Similarities between principal components of protein dynamics and random diffusion. Phys Rev E 62: 8438–8448. 10.1103/physreve.62.8438 11138145

[61] Hess B (2002) Convergence of sampling in protein simulations. Phys Rev E 65: 031910. 10.1103/PhysRevE.65.031910 11909112

[62] Simons KT, Kooperberg C, Huang E, Baker D (1997) Assembly of protein tertiary structures from fragments with similar local sequences using simulated annealing and Bayesian scoring functions. J Mol Biol 268: 209–225. 9149153 10.1006/jmbi.1997.0959

[63] Sivasubramanian A, Sircar A, Chaudhury S, Gray JJ (2009) Toward high-resolution homology modeling of antibody Fv regions and application to antibody-antigen docking. Proteins 74: 497–514. 19062174 10.1002/prot.22309 PMC2909601

[64] Chothia C, Lesk AM, Tramontano A, Levitt M, Smithgill SJ, et al (1989) Conformations Of Immunoglobulin Hypervariable Regions. Nature 342: 877–883. 2687698 10.1038/342877a0

[65] Kuroda D, Shirai H, Kobori M, Nakamura H (2009) Systematic classification of CDR-L3 in antibodies: implications of the light chain subtypes and the VL-VH interface. Proteins 75: 139–146. 18798566 10.1002/prot.22230

[66] Lensink MF, Mendez R, Wodak SJ (2007) Docking and scoring protein complexes: CAPRI 3rd Edition. Proteins 69: 704–718. 17918726 10.1002/prot.21804

[67] Mendez R, Leplae R, Lensink MF, Wodak SJ (2005) Assessment of CAPRI predictions in rounds 3-5 shows progress in docking procedures. Proteins 60: 150–169. 15981261 10.1002/prot.20551

[68] Paolella G, Caputo I, Marabotti A, Lepretti M, Salzano AM, et al (2013) Celiac anti-type 2 transglutaminase antibodies induce phosphoproteome modification in intestinal epithelial Caco-2 cells. PloS One 8: e84403. 24391952 10.1371/journal.pone.0084403 PMC3877280

[69] Caputo I, Lepretti M, Secondo A, Martucciello S, Paolella G, et al (2013) Anti-tissue transglutaminase antibodies activate intracellular tissue transglutaminase by modulating cytosolic Ca2+ homeostasis. Amino Acids 44: 251–260. 22038180 10.1007/s00726-011-1120-y

[70] Delano WL (2002) The PyMOL Molecular Graphics System. Available: http://www.pymol.org. Accessed 2014 July 1.

