# Supplementary material for: Structural Basis for the Recognition in an Idiotype-Anti-Idiotype Antibody Complex Related to Celiac Disease
Source: PLoS One. 2014 Jul 30;9(7):e102839. doi: 10.1371/journal.pone.0102839 (PMC4116137; doi:10.1371/journal.pone.0102839)
Supplement: File S1 — Supplementary Materials. Sequence S1, AIM2 DNA sequence. Sequence S2, AIM2 protein sequence. Table S1, list of inter-residue contacts in the 22 CDR solutions. Table S2, interface features of the six structure-known Ab1-Ab2 complexes. Table S3, list of 1DVF residues more than 50% buried upon complex formation. Table S4, cosine content of the first ten MD eigenvectors calculated for 1DVF and M1_CDR. Figure S1, intermolecular contact maps generated by COCOMAPS for the M1-CDR model and the six experimental structures available for Ab1-Ab2 complexes. Figure S2, RMSD fluctuation and Gyration radius, Rγ, for M1-CDR, M2-CDR, M1-blind and 1DVF along the MD simulations. Figure S3, LGA superimposition between the N-terminal domain of TG2 in its open form and the heavy chain of AIM2. (DOCX) [file pone.0102839.s001.docx]

Supplementary Materials for: “*Structural basis for the recognition in an idiotype-anti-idiotype antibody complex related to celiac disease*”, by Vangone *et al.*

**Sequence S1. AIM2 DNA sequence**

**VL sequence**

GATATTTTGCTCACTCAGTCTCCAGCAATCATGTCTGCGTCTCCAGGAgAGAAGGTCACCATGACCTGCAGTGCCAGCTCAAGTGTAAGTTACATGTACTGGTTCCAGCAgAAGCCAGGATCCTCCCCCAGACTCCTGATTTATGACACATCCAACCTGGCTTCTGGAGTCCCTGTTCGCTTCAGTGGCAGTGGGTCTGGGACCTCTTATTCTCTCACAATCAGCCGAATGGAGGCTGAAgATGCTGCCACTTATTACTGCCAGCAGTGGATTAGTTACCCGCTCACGTTCGGTGCTGGGACCAAGCTGGAGCTGAAACGT

**Linker sequence**

TCCGGAGGGTCGACCATAACTTCGTATAATGTATACTATACGAAGTTATCCTCGAGCGGTACC

**VH sequence**

CAGGTCCAGCTTCAGCAGTCTGGGGCTGAGCTTGTGAAGCCTGGGGCTTCAGTGAAGCTGTCCTGCAAGGCTTCTGGCTACACCCTCACCAGCTACTGGATGCAGTGGGTAAAACAGAGGCCTGGACAGGGCCTTGAGTGGATCGGAGAGATTGATCCTTCTGATAGCTATACTAACTACAATCAAAAGTTCAAGGGCAAGGCCACATTGACTGTAGACACATCCTCCAGCACAGCCTACATGCAGCTCAGCAGCCTGACATCTGAGGACTCTGCGGTCTATTACTGCGTTTACTACGGTAGTAGAGGGTTTGCTTACTGGGGCCAAGGGACTCTGGTCACTGTCTCCTCA

**Sequence S1. AIM2 protein sequence**

**VL sequence**

DILLTQSPAIMSASPGEKVTMTCSASSSVSYMYWFQQKPGSSPRLLIYDTSNLASGVPVRFSGSGSGTSYSLTISRMEAEDAATYYCQQWISYPLTFGAGTKLELKR

**Linker sequence**

SGGSTITSYNVYYTKLSSSGT

**VH sequence**

QVQLQQSGAELVKPGASVKLSCKASGYTLTSYWMQWVKQRPGQGLEWIGEIDPSDSYTNYNQKFKGKATLTVDTSSSTAYMQLSSLTSEDSAVYYCVYYGSRGFAYWGQGTLVTVSS

Table S1. Inter-residue contacts with conservation rates above 0.25 within the 22 CDR solutions. Contacts corresponding to H-bonds in M1-CDR are shaded.

| Ab1-MB2.8 | | Ab2-AIM2 |  | CR_kl_ |
| --- | --- | --- | --- | --- |
| Asp103_H | H3 | Ser97_H | H3 | 0.43 |
| Tyr94_L | L3 | Trp91_L | L3 | 0.39 |
| Lys50_L | L2 | Trp33_H | H1 | 0.39 |
| Trp32_L | L1 | Trp33_H | H1 | 0.39 |
| Asp103_H | H3 | Tyr95_H | H3 | 0.39 |
| Arg31_L | L1 | Asp54_H | H2 | 0.35 |
| Asp103_H | H3 | Gly96_H | H3 | 0.35 |
| Asp103_H | H3 | Trp33_H | H1 | 0.35 |
| Asp103_H | H3 | Tyr94_L | L3 | 0.35 |
| Tyr94_L | L3 | Tyr94_L | L3 | 0.30 |
| Lys50_L | L2 | Ser53_H | H2 | 0.30 |
| Lys50_L | L2 | Asp52_H | H2 | 0.30 |
| Trp32_L | L1 | Asp52_H | H2 | 0.30 |
| Arg31_L | L1 | Asp52_H | H2 | 0.30 |
| Thr104_H | H3 | Tyr94_L | L3 | 0.30 |
| Thr104_H | H3 | Trp33_H | H1 | 0.30 |
| Asp103_H | H3 | Trp91_L | L3 | 0.30 |
| Asp103_H | H3 | Arg98_H | H3 | 0.30 |
| Asp103_H | H3 | Tyr32_H | H1 | 0.30 |
| Tyr94_L | L3 | Arg98_H | H3 | 0.26 |
| Trp32_L | L1 | Tyr56_H | H2 | 0.26 |
| Arg31_L | L1 | Ser53_H | H2 | 0.26 |
| Arg31_L | L1 | Trp33_H | H1 | 0.26 |
| Thr105_H | H3 | Tyr94_L | L3 | 0.26 |
| Asp103_H | H3 | Tyr94_L | L3 | 0.26 |
| Ile102_H | H3 | Trp91_L | L3 | 0.26 |
| Ile102_H | H3 | Ser97_H | H3 | 0.26 |
| Arg59_H | H2 | Asp50_L | H2 | 0.26 |
| Trp33_H | H1 | Arg98_H | H3 | 0.26 |

**Table S2.** Some interface features for the six structure-known Ab1-Ab2 complexes.

| **PDB-ID** | **Interface**  **area (Å^2^)** | **# Interface**  **ress (Ab2/Ab1)** | **Polar contribution (%)** |
| --- | --- | --- | --- |
| 1cic | 771.05 | 44(24/20) | 61.5 |
| 1dvf | 816.4 | 46(21/25) | 45.6 |
| 1iai | 943.0 | 51(29/22) | 53.9 |
| 1pg7 | 1031.8 | 55(29/26) | 57.1 |
| 3bqu^a^ | 610.0 | 32(18/14) | 49.0 |
| Evans et al.^b^ | 1002.8 | 62(33/29) | 51.9 |
| *Average* | *862.5* | *49(26/23)* | *53.2* |

^a)^ This structure is anomalous because the heavy chain of Ab1 is incomplete.

^b)^ Structure not deposided in the PDB; coordinates courtesy of the authors: Evans, S.V., et al., *Exploring the mimicry of polysaccharide antigens by anti-idiotypic antibodies. The crystallization, molecular replacement, and refinement to 2.8 A resolution of an idiotope-anti-idiotope Fab complex and of the unliganded anti-idiotope Fab.* J Mol Biol, 1994. **241**(5): p. 691-705.

**Table S3.** 1DVF residues that are more than 50% buried upon complex formation.

| **E5.2-Ab2** | | **D1.3-Ab1** | |
| --- | --- | --- | --- |
| **Residue** | **Buried ASA(%)** | **Residue** | **Buried ASA(%)** |
| Tyr98_H | 95.24 | Glu98_H | 100.00 |
| Gln100_H | 91.33 | Gly33_H | 90.89 |
| Arg100b_H | 85.73 | Trp52_H | 87.63 |
| Gly100a_H | 84.54 | Gly53_H | 77.77 |
| Asp52_H | 70.20 | Trp92_L | 69.73 |
| Asn92_L | 61.81 | Tyr101_H | 69.64 |
| Tyr49_L | 61.09 | Tyr32_L | 68.47 |
| Thr93_L | 55.64 | Asp54_H | 62.52 |
| Ile97_H | 54.33 | Asn56_H | 59.32 |
|  |  | Gly31_H | 58.02 |
|  |  | Asp100_H | 56.90 |
|  |  | Arg99_H | 54.86 |
|  |  | Tyr49_L | 54.04 |
|  |  | Thr53_L | 51.74 |

**Table S4.** Cosine content of the first ten eigenvectors calculated for 1DVF and M1_CDR.

| **Eigenvector** | **1DVF** | **M1_CDR** |
| --- | --- | --- |
| 1 | 0.023 | 0.367 |
| 2 | 0.046 | 0.027 |
| 3 | 0.012 | 0.002 |
| 4 | 0.055 | 0.009 |
| 5 | 0.001 | 0.025 |
| 6 | 0.020 | 0.094 |
| 7 | 0.006 | 0.043 |
| 8 | 0.002 | 0.002 |
| 9 | 0.021 | 0.030 |
| 10 | 0.000 | 0.007 |


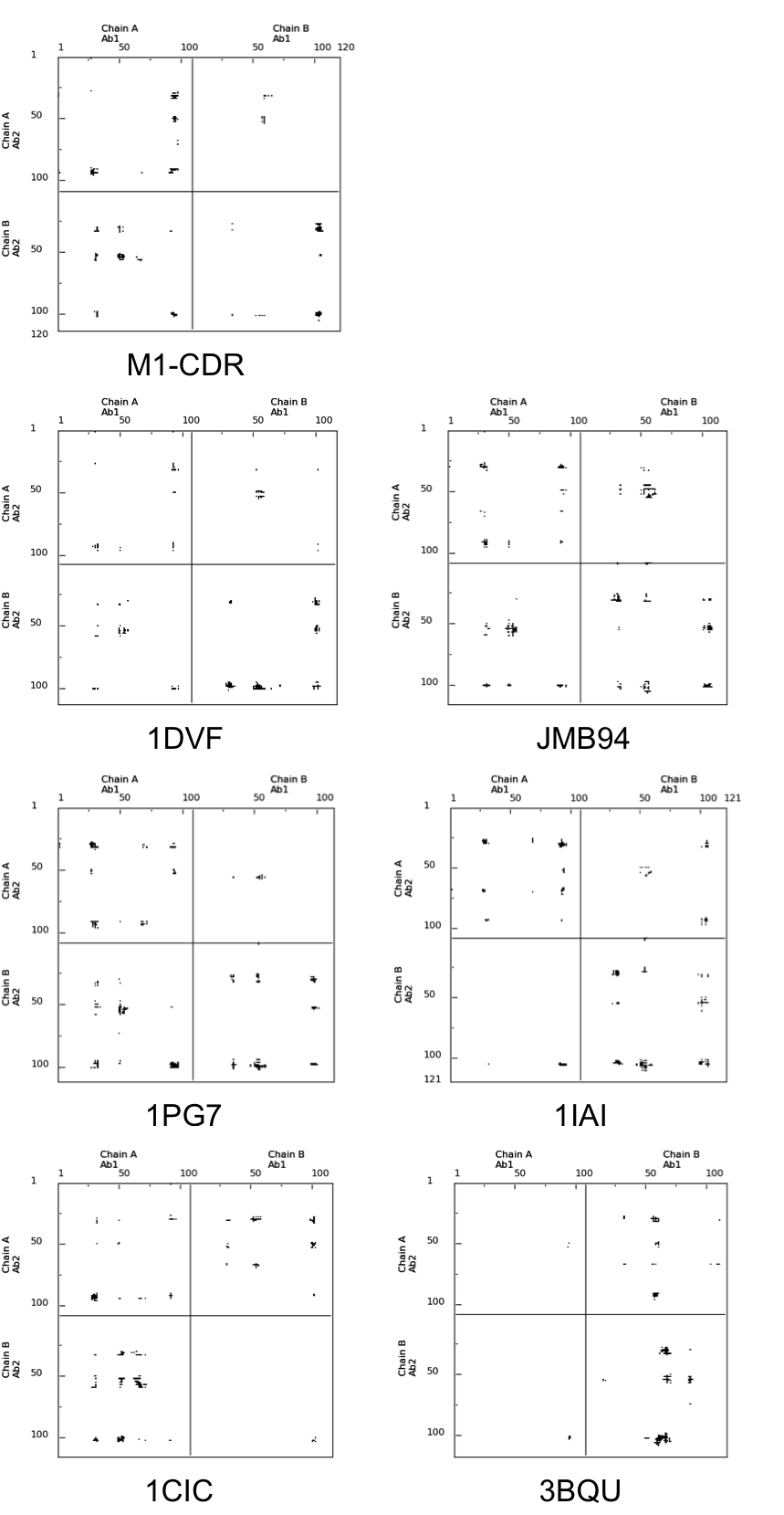


**Figure S1.** Intermolecular contact maps generated by COCOMAPS with the default distance treshold of 8 Å, for the M1-CDR model and the six experimental structures available for Ab1-Ab2 complexes. PDB codes are reported for 5 X-ray structures (1DVF, 1PG7, 1IAI, 1CIC and 3BQU) and our code (JMB94) is reported for the 6^th^ X-ray structure, published in JMB in 1994 (Evans et al., 1994, J Mol Biol 241:691) and never deposited in the PDB. In the Figure, light chains are named “A” and heavy chains “B”.

Contact maps of X ray structures in the middle of the figure (1DVF, JMB94, 1PG7 and 1IAI) are similar to that of the M1-CDR model, reflecting a similar binding mode. Contact maps of the two structures at the bottom of the Figure (1CIC and 3BQU) are instead significantly different from that of M1-CDR, reflecting a different binding mode. In particular, in 3BQU Ab1 participates to the binding only with its heavy chain, whereas in 1CIC contacts between the two heavy chains are absent, whereas contacts between the Ab1 heavy chain and the Ab2 light chain become important.


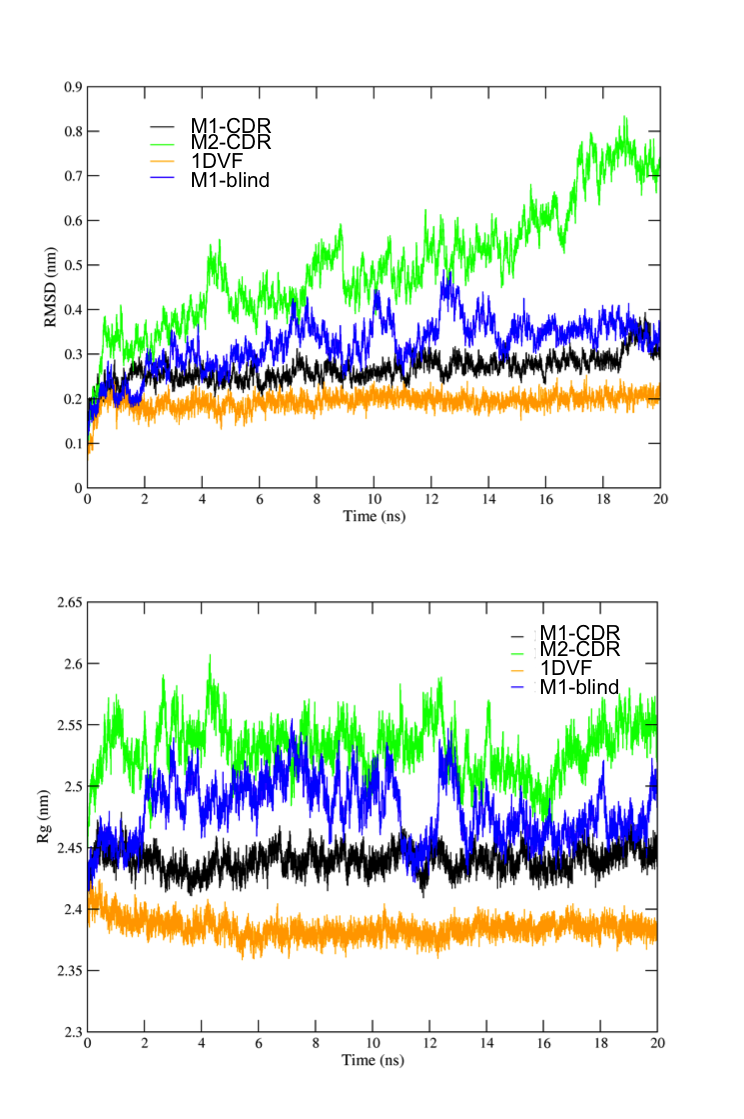


**Figure S2.** **Top**: RMSD fluctuation of the Cα atoms of M1-CDR, M2-CDR, M1-blind and 1DVF from the starting geometry along a 20-ns long MD trajectory. **Bottom**: Gyration radius, Rγ, of the systems along the simulation.


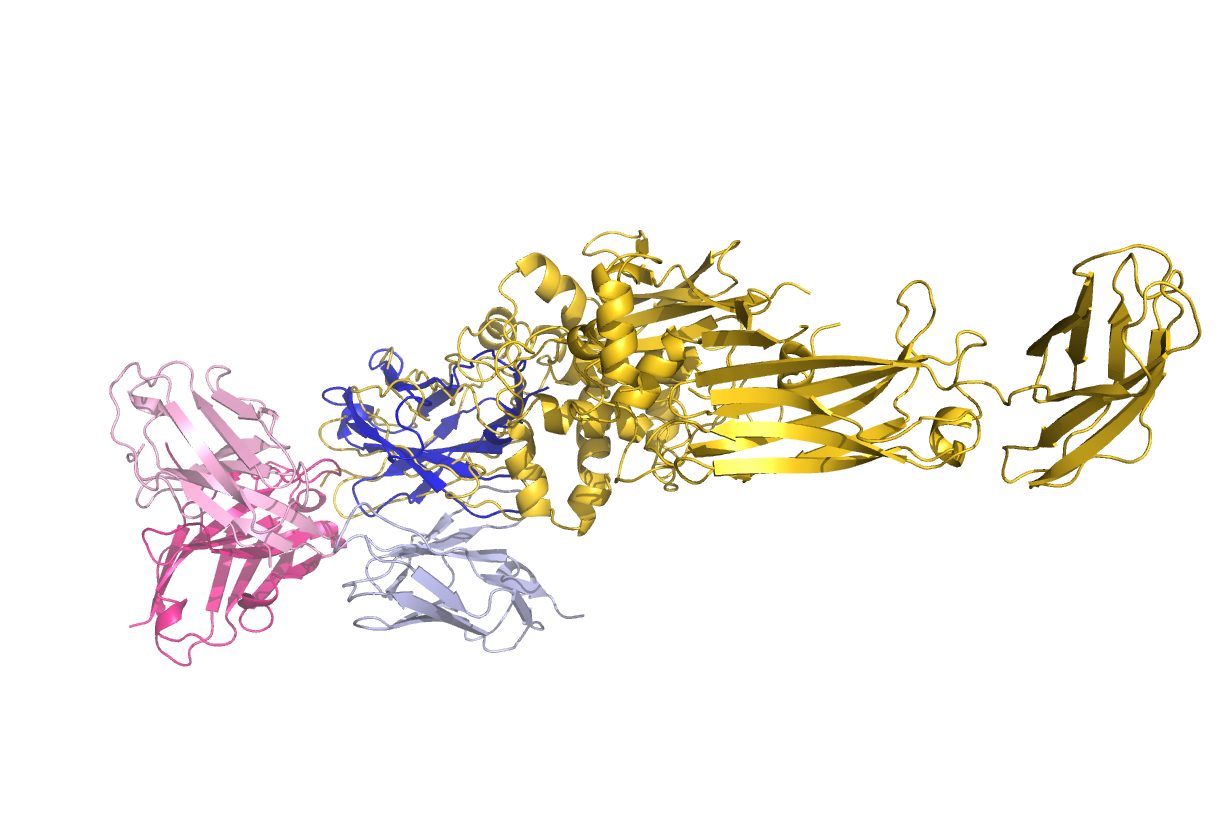


**Figure S3.** LGA superimposition between the N-terminal domain of TG2 in its open form, in gold (PDB ID: 2q3z), and the heavy chain of AIM2, in blue. AIM2 is shown bound to MB2.8 as in M1-CDR.
